# Basement Membrane–Related Genes C1QTNF3, PTGER2, CAMK2N1, PRSS36, and B3GNT7: Novel Biomarkers for Coronary Artery Disease

**DOI:** 10.1155/cdr/5358909

**Published:** 2026-07-09

**Authors:** Zhao Dong, Yuyu Zhang, Zhuang Li, Haozhen Yu, Wei Ge

**Affiliations:** ^1^ Department of General Practice, Xijing Hospital, Fourth Military Medical University, Xi′an, Shaanxi, China, fmmu.edu.cn; ^2^ Institute of Analytical Chemistry and Instrument for Life Science, the Key Laboratory of Biomedical Information Engineering of Ministry of Education, School of Life Science and Technology, Xi′an Jiaotong University, Xi′an, Shaanxi, China, xjtu.edu.cn

**Keywords:** basement membrane–related genes, coronary artery disease, key genes

## Abstract

**Background:**

Basement membrane–related genes (BMRGs) play a pivotal role in the pathogenesis of several diseases. However, their association with coronary artery disease (CAD) remains unexplored. Therefore, this investigation is designed to elucidate the involvement of BMRGs in CAD mechanisms.

**Methods:**

This study incorporated CAD‐related datasets (GSE113079 and GSE125856) and BMRGs. Initially, differentially expressed genes (DEGs) were identified between CAD and control (CTL) groups in GSE113079 and GSE125856. Subsequently, weighted gene coexpression network analysis (WGCNA) was carried out to identify key module genes associated with basement membranes. We then intersected the DEGs with the key module genes to obtain differentially expressed basement membrane–related genes (DE‐BMRGs). Two machine learning algorithms were applied to recognize feature genes, and the intersection of these features yielded the key genes. Subsequently, immune analysis, regulatory network construction, nomogram development, and expression verification were executed based on key genes.

**Results:**

Five key genes were identified: C1QTNF3, PTGER2, CAMK2N1, PRSS36, and B3GNT7. Among these, C1QTNF3 (AUC = 0.954), PTGER2 (AUC = 0.878), CAMK2N1 (AUC = 0.872), and B3GNT7 (AUC = 0.855) exhibited strong diagnostic potential in GSE113079, whereas PRSS36 showed more modest performance (AUC = 0.667). Moreover, 11 differences were observed in immune cell populations between the two groups (*p* < 0.05), including CD8 T cells, and we constructed a TF‐miRNA‐gene network comprising 112 regulatory relationships, such as the regulation of C1QTNF3 by SMAD4 and hsa‐miR‐302a‐3p. Additionally, the expression of these five key genes was consistently lower in the CAD group across GSE113079, GSE125856, and clinical patients.

**Conclusion:**

This study identified five key genes associated with the basement membrane (C1QTNF3, PTGER2, CAMK2N1, PRSS36, and B3GNT7) that offer valuable scientific insights into the mechanisms of CAD. These genes represent candidate noninvasive molecular markers requiring further validation for early diagnosis and serve as preliminary candidates for subsequent therapeutic target exploration.

## 1. Introduction

Coronary artery disease (CAD) is the most prevalent cardiovascular disorder worldwide, affecting approximately 197 million individuals and standing as a leading cause of disability and mortality [1]. CAD stems from atherosclerosis of the coronary arteries or atherosclerotic occlusion, leading to clinical manifestations like stable angina, unstable angina, myocardial infarction, and sudden cardiac death [2]. The underlying pathological process includes vascular and myocardial dysfunction triggered and exacerbated by factors such as ischemia, hypoxia, oxidative stress, inflammation, and apoptosis [3]. Early atherosclerosis stages involve the formation of fatty streaks in the intima, which induce a signal attracting smooth muscle cells to the site, followed by smooth muscle cell proliferation, extracellular matrix production, and the development of atherosclerotic plaques. These plaques accumulate abundant extracellular matrix, ultimately leading to significant coronary artery stenosis or occlusion [2]. Currently, the treatment of CAD relies on conservative therapy, including antithrombotic agents, lipid‐modulating drugs, and anti‐ischemic medications combined with lifestyle interventions. While these approaches can alleviate symptoms, they do not directly address the underlying organic lesions. Percutaneous coronary intervention, which involves stent implantation to relieve stenosis, is suitable for acute myocardial infarction but does not significantly improve the prognosis of chronic CAD and carries a risk of restenosis [4, 5]. Coronary artery bypass grafting is more effective for complex multivessel disease but is associated with higher surgical risks. Moreover, limitations also exist in the diagnostic realm. Coronary angiography, while considered the gold standard for diagnosing CAD, is an invasive procedure associated with high costs and risks of complications such as restenosis or thrombosis, making it unsuitable for widespread early stage screening [6]. In this context, the identification of noninvasive biomarkers holds great promise for improving early warning, risk stratification, and dynamic monitoring of CAD, potentially enabling interventions that delay, halt, or even reverse disease progression [7]. The development of next‐generation CAD diagnostic tools therefore necessitates the integration of multifaceted technical approaches, including molecular biomarkers. Given these limitations in both therapy and early diagnosis, a deeper exploration of the molecular mechanisms underlying CAD pathogenesis is essential to identify novel diagnostic markers and therapeutic targets.

Dynamic remodeling of the extracellular matrix is a hallmark of atherosclerotic plaque progression. Basement membranes (BMs), specialized structures underlying endothelial cells, are vital for vascular wall integrity and function. Genetic alterations in BM components can therefore lead to a range of pathological conditions [8–10]. There is growing evidence that BMs are critically involved in the progression of CAD through processes such as inflammation, metabolic dysregulation, and impaired vascular function. For instance, in acute coronary syndromes associated with plaque erosion, injury to the BM contributes to disease pathogenesis by disrupting endothelial cell anchorage and promoting thrombosis [11]. Damage to BMs amplifies the local inflammatory response. This process shares similarities with other vascular pathologies in which NLRP3 inflammasome activation drives tissue injury [12]. Moreover, metabolic disturbances—including dyslipidemia and oxidative stress—further exacerbate BM dysfunction, which compromises vascular homeostasis and accelerates plaque progression [13]. Furthermore, polymorphisms in the COL4A1 gene, which encodes the core BM component Type IV collagen, are significantly associated with increased CAD susceptibility, suggesting that compromised BM integrity is an early event in disease onset [14]. Additionally, matrix metalloproteinase (MMP)–derived degradation products of BM components, such as Type IV collagen, have been identified as potential biomarkers for cardiovascular events [15]. Collectively, these findings position BMs not merely as passive structural elements but as active contributors to CAD pathogenesis. We therefore hypothesize that BM‐related genes hold significant pathophysiological importance in CAD and represent potential diagnostic and therapeutic targets. Nevertheless, a comprehensive analysis specifically targeting basement membrane–related genes (BMRGs) in CAD is still lacking. To our knowledge, this study represents the first exploratory effort to systematically characterize the expression profiles, diagnostic potential, and underlying regulatory networks of BMRGs in CAD.

In this current study, we identified pivotal genes linked to the BM through the mining of Gene Expression Omnibus (GEO) data and various bioinformatics methods. These genes were then examined for their relevance as potential biomarkers for CAD, with an exploration of the underlying regulatory mechanisms. This endeavor may lay the theoretical groundwork for more comprehensive CAD research.

## 2. Materials and Methods

### 2.1. Data Sources

The CAD‐associated datasets (GSE113079 and GSE125856) were obtained from the GEO repository (https://www.ncbi.nlm.nih.gov/gds). GSE113079 included 93 CAD blood samples and 48 control (CTL) samples. GSE125856 comprised tissue samples from 18 CAD patients and 12 CTL individuals, including epicardial adipose tissue (EAT), subcutaneous adipose tissue (SAT), and mediastinal adipose tissue (mAT). In this study, we retrieved 222 BMRGs from the Basement Membrane Database (bmBASE, available at https://bmbase.manchester.ac.uk/) [16].

### 2.2. Differential Analysis

Differentially expressed genes (DEGs) between CAD and CTL samples in datasets GSE113079 and GSE125856 were identified using the limma package, with cutoff criteria of |log2FC| > 0.5 and *p* < 0.05 [17].

### 2.3. Screening of Key Module Genes

Initially, using 222 BMRGs as the feature gene set, we computed individual BM scores for all GSE113079 samples through the single‐sample gene set enrichment analysis (ssGSEA) method in the GSVA package [18]. The weighted gene coexpression network analysis (WGCNA), implemented with the WGCNA package, was performed, followed by sample clustering to remove outliers. Then, the optimal soft threshold was set based on the scale‐free fitting index (signed *R* ^2^) and the mean connectivity (close to 0). Coexpression network modules were extracted using dynamic tree cutting with the following parameters: minimum module size = 100 genes and module merging parameter = 0.2 [19]. Modules showing strong correlation with the BM score were designated as key modules (|correlation (cor)| > 0.3, *p* < 0.05) [20].

### 2.4. Identification of Differentially Expressed (DE) BMRGs and Functional Analysis

DE‐BMRGs were determined by the intersection of DEGs from GSE113079 (DEGs1), DEGs from GSE125856 (DEGs2) and key module genes from the key modules. Enrichment analysis was conducted using the clusterProfiler package and the human gene annotation package org.Hs.eg.db, referencing Gene Ontology (GO) and Kyoto Encyclopedia of Genes and Genomes (KEGG) databases (*p* value < 0.05) [21].

### 2.5. Machine Learning

DE‐BMRGs were subjected to feature selection through both LASSO (glmnet) and SVM‐RFE (caret) algorithms to identify key biomarker candidates [22]. In the LASSO regression analysis, 10‐fold cross‐validation was performed. The selection of genes was determined when the regularization parameter (lambda) reached its minimum value. In the SVM‐RFE analysis, the feature ranking process was accomplished within the framework of fivefold cross‐validation. Features that contributed the least to the model were iteratively removed to identify the optimal feature subset. The consensus genes derived from the intersection of both feature selection methods were identified as core biomarkers. We quantified the diagnostic power of these core genes in GSE113079 and GSE125856 through ROC analysis implemented in the pROC environment [23].

### 2.6. Immune Analysis

Immune cell infiltration profiles were quantified using CIBERSORT, estimating the relative proportions of 22 immune cell subtypes across GSE113079 samples. The differences in 22 types of immune cells between CAD samples and CTL samples were compared by the Wilcoxon rank‐sum test (*p* < 0.05). Subsequent Spearman′s correlation analysis evaluated associations among immune cell populations, key genes, and intercellular relationships (|cor| > 0.3, *p* < 0.05). The *p* values were adjusted using the false discovery rate (FDR) method.

### 2.7. Construction of Regulatory Network

To predict miRNAs targeting key genes, multiple databases, such as miRanda (http://mirtarbase.mbc.nctu.edu.tw), miRDB (http://www.mirdb.org/), and miCrocosm, were utilized by incorporating the multiMiR package. Subsequently, potential transcription factor (TF) regulators of the identified key genes were predicted through the NetworkAnalyst platform (https://www.networkanalyst.ca/). Ultimately, we constructed miRNA‐gene networks, TF‐gene networks, and TF‐miRNA‐gene networks using Cytoscape [24].

### 2.8. Creation of Nomogram

In GSE113079 and GSE125856, a nomogram containing key genes was created with the “rms” package [25].

First, a binary logistic regression model was constructed using key genes as independent variables and disease status as the dependent variable, following the formula below:

Logit P=β0+β1×gene1+β2×gene2+⋯+βn×genen.

*P* denotes the probability of an individual developing the disease. *β*_0_ represents the intercept term. *β*_0_ to *β*_*n*_ stand for the regression coefficients of the genes, which are obtained by fitting the data using the glm() function.

The formula for risk probability conversion is as follows:

P=11+e−logit P.

In the nomogram, the “total points” axis is parallel to the “risk probability” axis. The corresponding risk probability for a given total score can be quickly queried by connecting these two axes with a vertical line, and a higher total score indicates a higher risk probability.

To evaluate the predictive capacity of the model, calibration curves, decision curve analysis (DCA), and clinical impact curve (CIC) were employed.

### 2.9. Blood Samples

Blood samples collected from patients with coronary heart disease (CAD group) and matched healthy individuals (CTL group) in Xijing Hospital, between November and December 2023, were used for the purpose of verifying differential gene expression. The study was conducted in accordance with the Declaration of Helsinki and approved by the Medical Ethics Committee of Xijing Hospital (Ethics Review Number: KY20232338‐C‐1) for studies involving humans, and all patients signed informed consent.

### 2.10. Preliminary Experimental Verification of Key Gene Expression by qRT‐PCR

This preliminary validation was performed in a small independent clinical cohort, with only six CAD patients and six age‐ and gender‐matched healthy CTLs included, aiming to preliminarily verify the differential expression patterns of key genes at the transcriptional level. Blood samples were obtained from CAD patients and CTLs (*n* = 6 per group) for quantitative real‐time PCR (qPCR). Total RNA isolation was performed using the Blood Total RNA Extraction Kit (Servicebio, Wuhan, China). RNA concentration and purity were assessed with a NanoDrop 2000 spectrophotometer (Thermo Fisher Scientific, United States). All samples yielded A260/A280 ratios between 1.8 and 2.1, indicating high‐purity RNA. Integrity was then evaluated by 1.5% agarose gel electrophoresis, which revealed sharp 28S and 18S rRNA bands with an intensity ratio of approximately 2:1, confirming intact RNA. cDNA was then synthesized from the qualified RNA, and each cDNA sample was analyzed in triplicate. Gene‐specific primers spanning exon–exon junctions were designed using Primer‐BLAST (https://www.ncbi.nlm.nih.gov/tools/primer-blast/). Quantitative PCR was performed in 25‐*μ*L reactions containing 10 ng cDNA template using FastStart Universal SYBR Green Master mix (Takara Biomedical Technology [Beijing] Co.) on a CFX96TM Real‐Time System (Bio‐Rad), with GAPDH serving as the endogenous control. Refer to Table 1 for the primer sequences.

**Table 1:** Primer sequences.

Primer name	Sequence (5 ^′^‐3 ^′^)
*C1QTNF3* (H)‐F	GCCCCAGTATCAGGTGTGTA
*C1QTNF3* (H)‐R	GCAAAGGTGGAGAAGCGTTG
*PTGER2* (H)‐F	GCTCCTTGCCTTTCACGATTT
*PTGER2* (H)‐R	AGGATGGCAAAGACCCAAGG
*CAMK2N1* (H)‐F	CGGAGCAAGCGGGTTGTTAT
*CAMK2N1* (H)‐R	CACAACAGACTGCAAGGGGA
*PRSS36* (H)‐F	AGACCCAGTCAGATCCCCAG
*PRSS36* (H)‐R	CAACGCGAGTCATTCTCCTCCT
*B3GNT7* (H)‐F	GCCATGTCGCTGTGGAAGA
*B3GNT7* (H)‐R	ACCAGCTGTCCATTTGGCTT
*GAPDH* (H)‐F	GGTGAAGGTCGGAGTGAACG
*GAPDH* (H)‐R	CTCGCTCCTGGAAGATGGTG

### 2.11. Statistical Analysis

All data processing and statistical analyses were conducted using R software. Intergroup comparisons were performed using either *t*‐tests or Wilcoxon rank‐sum tests, as appropriate. Quantitative data were analyzed with GraphPad Prism 9.0 and expressed as mean ± SEM. Statistical significance was defined as *p* < 0.05.

## 3. Results

### 3.1. The DEGs Between CAD and CTL Were Identified

**Figure 1:**
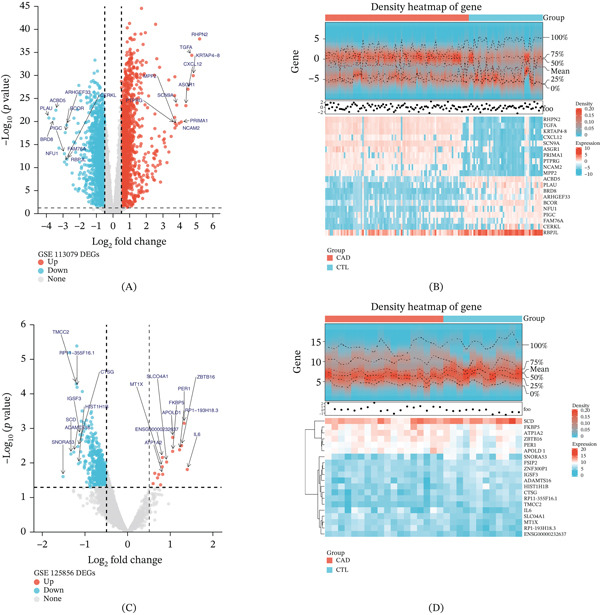
Identification of differentially expressed genes (DEGs) in CAD and CTL. (A, C) Volcano plots for DEGs in CAD and CTL samples from (A) GSE113079 and (C) GSE125856. Red dots denote upregulated genes, whereas blue dots signify downregulated genes in CAD samples; gray indicates genes with no significant changes. The *x*‐axis (log_2_ fold change) denotes the log_2_‐transformed fold change of gene expression between the two comparison groups, reflecting the magnitude and direction of differential expression, and the *y*‐axis (−log_10_(*p*value)) represents the negative base‐10 logarithm of the *p* value for each gene′s differential expression, which reflects the statistical significance of the expression change. (B) A heatmap displaying the distribution of DEGs in CAD (in red) and CTL (in blue) from GSE113079. (D) A heatmap showing the DEGs in CAD (in red) and CTL (in blue) from GSE125856.

In GSE113079, a total of 3625 DEGs1 were identified, comprising 1711 upregulated and 1914 downregulated (Figure 1A,B). In GSE125856, there were 653 DEGs2, with 15 genes upregulated and 638 genes downregulated (Figure 1C,D).

### 3.2. A Total of 2910 Key Module Genes Were Screened

**Figure 2:**
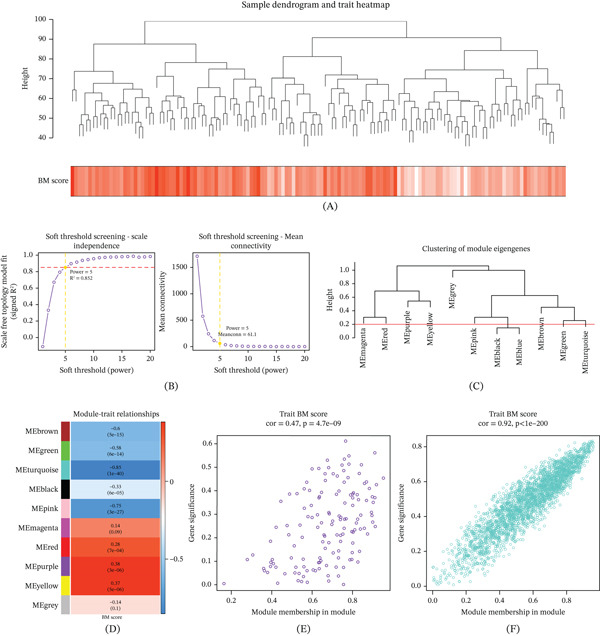
Screening of module genes relevant to BM score in GSE113079. (A) Sample clustering and phenotypic heatmap. (B) Screening for optimal soft threshold. The left panel: The *x*‐axis represents the soft threshold, and the *y*‐axis represents the fitting index corresponding to each soft threshold. The right panel: The *x*‐axis represents the soft threshold, and the *y*‐axis represents the average connectivity corresponding to each soft threshold. (C) Creation of a coexpression network. (D) Correlation heatmap between modules and BM score. (E, F) Correlation between gene significance (GS) and module membership (MM) in the (E) MEpurple module and the (F) MEturquoise module.

No outliers were observed in CAD samples from GSE113079 (Figure 2A). The analysis of fitting indices and mean connectivity across different soft thresholds indicated that a power of *β* = 5 was optimal, as it was the lowest value at which the scale‐free topology fit index (*R* ^2^) first exceeded 0.85, whereas the mean connectivity remained relatively high (Figure 2B, Supporting Information 1: Table S1). Subsequently, 10 coexpression modules were established (Figure 2C). Among these, MEpurple (cor = 0.38, *p* value = 3e − 06) and MEturquoise showed the strongest correlations with BM scores (cor = −0.85, *p* value = 1e − 40) (Figure 2D). These two modules were designated as key modules, including a total of 2910 key module genes (Figure 2E,F).

### 3.3. DE‐BMRGs Were Enriched in the Glucose Metabolic Process and the Pentose Phosphate Pathway (PPP)

**Figure 3:**
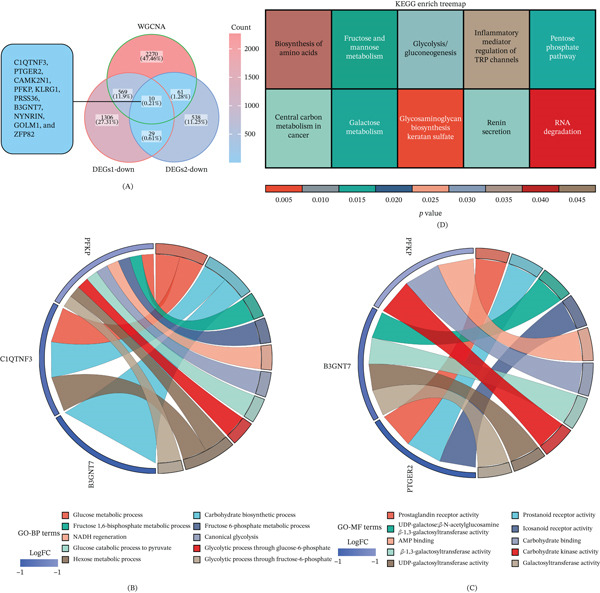
Identification of DE‐BMRGs and their functional enrichment in CAD. (A) Venn diagram depicting the 10 common genes. (B) Chord diagram illustrating the GO‐BP enrichment of DE‐BMRGs. (C) Chord diagram demonstrating the GO‐MF enrichment of DE‐BMRGs. (D) Treemap illustrating the KEGG enrichment of DE‐BMRGs.

By intersecting DEGs1, DEGs2, and key module genes, we identified 10 DE‐BMRGs: C1QTNF3, PTGER2, CAMK2N1, PFKP, KLRG1, PRSS36, B3GNT7, NYNRIN, GOLM1, and ZFP82 (Figure 3A). In biological processes (BPs), these DE‐BMRGs were enriched in processes like glucose metabolism, carbohydrate biosynthesis, and fructose 1,6‐bisphosphate metabolism (Figure 3B). In terms of molecular function (MF), the DE‐BMRGs were associated with prostaglandin receptor activity and prostanoid receptor activity (Figure 3C). Additionally, the DE‐BMRGs were implicated in the PPP and galactose metabolism, among others, as indicated in the KEGG enrichment analysis (Figure 3D). These enrichment results shed light on the potential roles of DE‐BMRGs in metabolic reprogramming and signaling transduction, laying a foundation for exploring their regulatory mechanisms in the research context.

### 3.4. C1QTNF3, PTGER2, CAMK2N1, PRSS36, and B3GNT7 Were Defined as Key Genes

**Figure 4:**
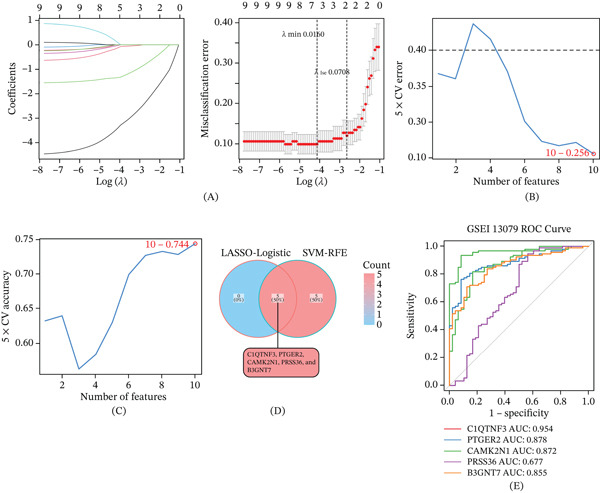
Screening of key genes using LASSO regression and SVM‐RFE in GSE113079. (A) Five genes identified via the LASSO model. The left panel is a coefficient penalty plot for logistic regression: The *x*‐axis represents the logarithmic value of the regularization parameter lambda, and the *y*‐axis represents the regression coefficient values of the feature variables. Each curve depicts the variation trajectory of a gene′s regression coefficient across different lambda values. The right panel is a cross‐validation error curve plot: The *x*‐axis represents the logarithmic value of lambda, and the *y*‐axis represents the partial likelihood deviance. The dashed line indicates the optimal regularization parameter selected via cross‐validation, and each point denotes the number of variables included in the model under different regularization parameters. (B) Error rate change curve. The *x*‐axis represents the number of features, and the *y*‐axis represents the error rate. (C) Accuracy change curve. The *x*‐axis represents the number of features, and the *y*‐axis represents the accuracy. (D) Venn diagram showing the intersection of five genes. (E) ROC curve illustrating the performance of key genes in GSE113079. The *x*‐axis represents the false‐positive rate (FPR), and the *y*‐axis represents the true‐positive rate (TPR).

In GSE113079, five feature genes were identified through LASSO analysis, which included C1QTNF3, PTGER2, CAMK2N1, PRSS36, and B3GNT7 (Figure 4A). Furthermore, 10 feature genes were extracted using SVM‐RFE, including C1QTNF3, PTGER2, CAMK2N1, PFKP, KLRG1, PRSS36, B3GNT7, NYNRIN, GOLM1, and ZFP82 (Figure 4B,C). The intersection of these feature genes derived from two machine learning algorithms yielded five key genes: C1QTNF3, PTGER2, CAMK2N1, PRSS36, and B3GNT7 (Figure 4D). It is worth noting that, except for PRSS36, the AUC values of the other key genes exceeded 0.7 in GSE113079, highlighting their potential diagnostic value (Figure 4E). In the validation dataset GSE125856, the AUC values were as follows: C1QTNF3 = 0.648, PTGER2 = 0.708, CAMK2N1 = 0.775, PRSS36 = 0.750, and B3GNT7 = 0.699 (Supporting Information 2: Figure S1). Among these, CAMK2N1 and PRSS36 showed AUCs > 0.75, indicating promising diagnostic potential, whereas the others demonstrated moderate performance. Notably, significant tissue heterogeneity exists between the two datasets: GSE113079 was derived from peripheral blood samples, whereas GSE125856 included EAT, SAT, and mAT samples. Tissue‐specific gene expression patterns inevitably lead to differences in the expression levels and diagnostic performance of the five key genes between datasets, which is an important factor to be considered when interpreting the external validation results. Further validation in larger, tissue‐matched cohorts is warranted.

### 3.5. There Were 11 Different Immune Cells in GSE113079

**Figure 5:**
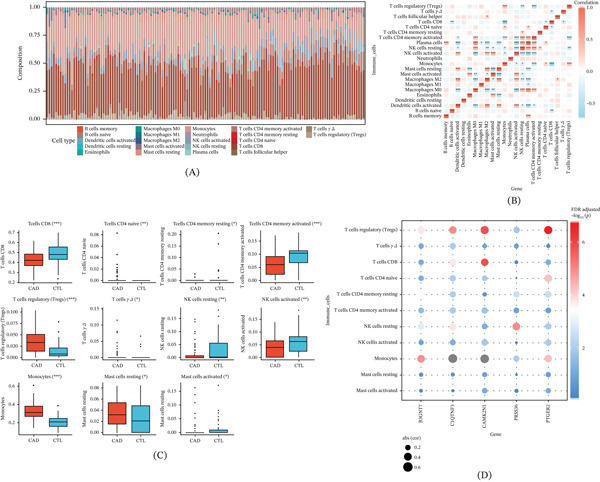
Estimation of immune cell abundance using the CIBERSORT algorithm. (A) Histogram displaying the infiltration abundance of different immune cell types via the CIBERSORT algorithm. (B) Heatmap illustrating the correlation between various immune cell types ( ^∗^ *p* < 0.05,  ^∗∗^ *p* < 0.01, and  ^∗∗∗^ *p* < 0.001). (C) Boxplots demonstrating immune cell abundance across different groups ( ^∗^ *p* < 0.05,  ^∗∗^ *p* < 0.01, and  ^∗∗∗^ *p* < 0.001; CAD vs. CTL). The *x*‐axis represents different groups, and the *y*‐axis represents the infiltration abundance of immune cells in each group. (D) Heatmap depicting the correlation between five key genes and different immune cell types.

We estimated the abundance of 22 immune cell types in all samples from GSE113079 (Figure 5A). Spearman correlation analysis (Figure 5B) revealed the interactions among these immune cells. Notably, comparisons between CAD and CTL groups revealed significant differences in the abundances of 11 distinct immune cell types, including CD8 T cells, activated CD4 memory T cells, regulatory T cells (Tregs), resting NK cells, and monocytes (Figure 5C). Furthermore, we observed strong positive correlations between CAMK2N1 and CD8 T cells, as well as strong negative correlations between CAMK2N1 and monocytes (|cor| > 0.4, *p* *v* *a* *l* *u* *e* < 0.05) (Figure 5D). These findings imply that CAMK2N1 might modulate CAD‐associated immune dynamics by interacting with CD8 T cells and monocytes, providing clues for exploring CAD′s immunological mechanisms.

### 3.6. The Construction of a Regulatory Network With Key Genes

**Figure 6:**
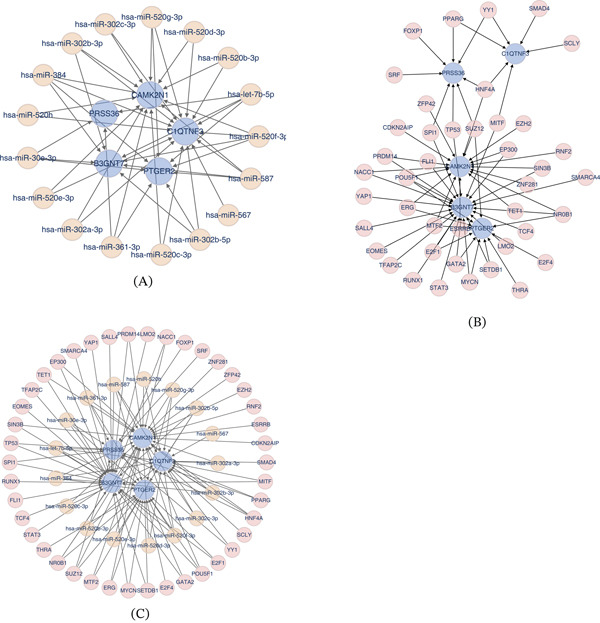
Construction of regulatory networks for key genes. (A) miRNA‐mRNA regulatory network for key genes. (B) TF‐mRNA regulatory network for key genes. (C) TF‐mRNA‐miRNA regulatory network for key genes. Blue represents key genes; orange represents miRNA; pink represents TF.

In Figure 6A, the miRNA‐gene network is shown, where, for example, hsa‐miR‐302b‐5p and hsa‐miR‐361‐3p are targeted by PTGER2. In Figure 6B, the TF‐gene network is established, with C1QTNF3 being simultaneously regulated by SCLY, SMAD4, YY1, and peroxisome proliferator–activated receptor gamma (PPARG), as illustrated. Figure 6C displays the TF‐miRNA‐gene network, which comprises 112 relational pairs, such as C1QTNF3 being regulated by SMAD4 and hsa‐miR‐302a‐3p. These multilevel regulatory networks clarify the intricate interactions between TFs, miRNAs, and key genes, providing a clear framework for further exploring core regulatory targets in the research scenario.

### 3.7. The Nomogram Model Had the Ability of Accurate Prediction

**Figure 7:**
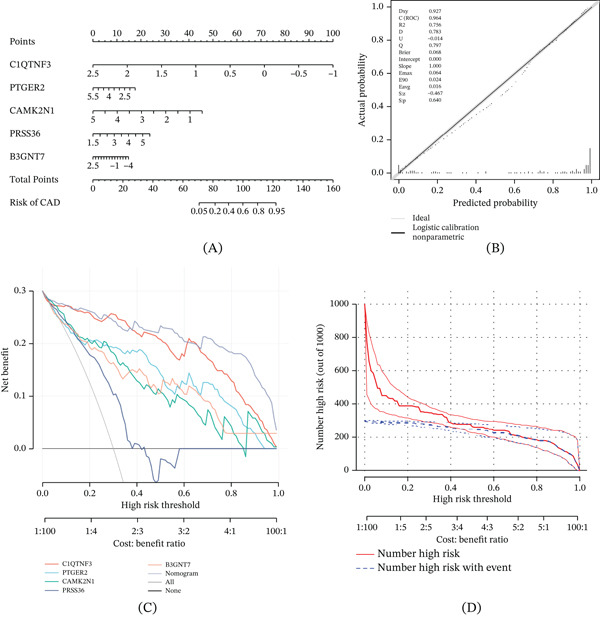
Construction and validation of the nomogram model. (A) The nomogram model constructed by five key genes. (B) Calibration curve for assessing the predictive capability of the nomogram model. The *x*‐axis represents the predicted probability of the event occurrence by the model, and the *y*‐axis represents the actual observed probability of the event occurrence. (C) DCA curve for evaluating the clinical applicability of the nomogram models. The *x*‐axis represents the high‐risk threshold, and the *y*‐axis represents the net benefit. (D) Clinical impact curve of the nomogram models. The *x*‐axis represents the high‐risk threshold, and the *y*‐axis represents the number high risk, which indicates the count of individuals classified as high risk.

A nomogram model was created to determine the risk rate for CAD patients, as depicted in Figure 7A. Calibration curves (Figure 7B) showed that the model had a slope close to 1, indicating its accuracy. Furthermore, the DCA illustrated the clinical benefit of the model compared to individual genes (Figure 7C). The CIC revealed that the model performed well in predicting CAD risk (Figure 7D). The same results were obtained in GSE125856 (Supporting Information 3: Figure S2), further suggesting the preliminary diagnostic potential of these candidate genes for CAD.

### 3.8. Preliminary qRT‐PCR Validation Confirmed Downregulation of Five Key Genes in CAD Samples

**Figure 8:**
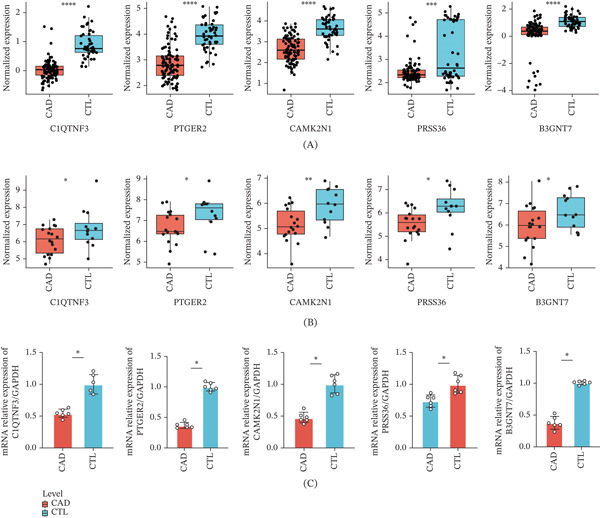
Basement membrane‐related gene expression. (A) GSE113079 boxplot. (B) GSE125856 boxplot, CAD vs. CTL. (C) Preliminary qRT‐PCR verification for five key genes. The x‐axis represents different groups, and the y‐axis represents the expression level of each key gene in the groups. Asterisks indicate statistical significance:  ^∗^ *p* < 0.05,  ^∗∗^ *p* < 0.01,  ^∗∗∗^ *p* < 0.001,  ^∗∗∗∗^ *p* < 0.0001.

Furthermore, we observed downregulation of five key genes in the CAD group of both GSE113079 (Figure 8A) and GSE125856 (Figure 8B). To experimentally validate the transcript levels of these key genes, we performed qRT‐PCR on blood samples from both healthy CTLs and CAD patients. The expression patterns of these five genes in this small clinical cohort were largely consistent with the dataset validation results (Figure 8C), though confirmation in larger cohorts is warranted.

## 4. Discussion

CAD is a prevalent cardiovascular disorder worldwide and a leading cause of morbidity and mortality [1]. While BMs have been implicated in CAD pathogenesis [11, 14], the role of BMRGs remains unclear. To address this, our bioinformatics analysis identified five key BMRGs in CAD and investigated their underlying mechanisms, thereby providing novel insights for CAD research.

Functional enrichment analysis revealed that the identified DE‐BMRGs were significantly enriched in the PPP. The PPP serves as the primary source of nicotinamide adenine dinucleotide phosphate (NADPH), a key cofactor for maintaining cellular antioxidant systems, such as glutathione, to counteract oxidative stress [26, 27]. Oxidative stress plays a multifaceted and critical role in the pathophysiology of CAD [28]: It directly impairs endothelial cells, promotes foam cell formation via the oxidation of low‐density lipoprotein (ox‐LDL), and activates proinflammatory signaling pathways [29, 30]. Furthermore, oxidative stress can activate MMPs, whose activity is crucial for BM degradation [31]. An impaired PPP may lead to NADPH deficiency and exacerbated oxidative stress, thereby potentially promoting BM degradation and plaque destabilization. Our findings indicate an association between BMRGs and the PPP, suggesting that BMRG dysregulation in CAD may compromise vascular antioxidant defenses, amplifying oxidative stress–induced endothelial dysfunction and plaque instability. The DE‐BMRGs were also enriched in glucose metabolism, a pathway strongly implicated in CAD development and progression. Dysregulation of glucose metabolism has been shown to play an important role in the onset and progression of CAD. Under different metabolic conditions, glycolipid biomarkers exhibit varying predictive efficacy for CAD [32]. Another study indicated that glucose metabolic status acts as an effect modifier in the relationship between the triglyceride–glucose (TyG) index and in‐hospital mortality among CAD patients, with the TyG index showing a more pronounced predictive value for mortality risk in diabetic individuals [33]. In addition, several signaling pathways have been implicated in CAD progression. For instance, the RhoA/ROCK‐1 pathway influences CAD pathogenesis by modulating cellular and vascular functions, and inhibition of this pathway may offer therapeutic benefits [34]. Meanwhile, the IFN‐*γ*/JAK2/STAT1 signaling pathway promotes inflammation and pyroptosis via AIM2 inflammasome activation, thereby contributing to CAD development [35]. Collectively, these findings highlight the significant roles of glucose metabolism dysregulation and multiple signaling pathways in CAD pathogenesis, offering new perspectives and potential targets for early diagnosis and targeted therapy.

C1QTNF3, also known as CTRP3, is a member of the C1q/TNF‐associated protein family [36]. It is a secreted protein with structural similarities to lipocalins and is expressed in various tissues and organs, including the heart, bone, and kidney. Clinical studies have shown that serum CTRP3 levels are reduced in individuals at a high risk of CAD, such as those who are obese, hypertensive, or diabetic. Additionally, CTRP3 levels in CAD patients are correlated with the severity of coronary artery lesions [37]. C1QTNF3 plays a protective role in the cardiovascular system by promoting angiogenesis, enhancing ventricular remodeling following myocardial infarction, and improving the function of damaged endothelial cells and cardiomyocytes [38]. Therefore, C1QTNF3 may influence CAD by promoting angiogenesis, enhancing postinfarction ventricular remodeling, and improving the function of damaged endothelial cells and cardiomyocytes. Notably, its expression is downregulated in CAD.

The PTGER2 gene is a receptor for prostaglandin E2 (PGE2). Acting as a stimulatory G protein–coupled receptor, PGE2 binding leads to an increase in intracellular cAMP levels and is involved in various physiological and pathological processes in the human body. PTGER2 can be expressed in a wide range of cell types, including cardiomyocytes, fibroblasts, and endothelial cells. Studies in mice have shown that overexpression of PTGER2 or the application of EP2 receptor agonists can inhibit neovascular endothelial restenosis and the migration of vascular smooth muscle cells induced by PDGF, in a mouse model. CAD patients with mutations in the PTGER2 gene are at an increased risk of developing acute coronary syndromes. The PGE2‐EP2 receptor signaling also promotes cardiomyocyte differentiation after myocardial infarction. However, some studies have reported that EP2 receptor expression is elevated in atherosclerotic plaques, potentially contributing to the progression of atherosclerosis [39]. Therefore, the role of the EP2 receptor, the PTGER2 gene product, in CAD still requires further investigation. The role of PTGER2 in CAD remains controversial, with studies reporting seemingly opposing effects. On one hand, activation of the EP2 receptor by PGE2 elevates intracellular cAMP levels, promoting the recruitment of monocytes/macrophages into the subendothelial space and thereby contributing to atherosclerosis‐related inflammation [40]. However, other evidence suggests that PTGER2 can exert antiatherosclerotic effects by inhibiting abnormal vascular smooth muscle cell proliferation, reducing endothelial injury, and modulating lipid metabolism [41]. Given that our study observed downregulation of PTGER2 in CAD alongside its correlation with immune cells (e.g., CD8+ T cells and monocytes), we speculate that this discrepancy may be model‐dependent. The observed downregulation may primarily impair its protective functions—such as restraining vascular smooth muscle cell migration and maintaining BM integrity—while its potential proinflammatory role might not be dominant at this specific disease stage. In summary, the function of PTGER2 appears to be dual‐phase. During early disease stages, its anti‐inflammatory and endothelial‐protective effects likely predominate. However, in advanced stages of plaque formation, cross‐activation of other signaling pathways may shift its role toward promoting plaque progression. This model requires further validation through larger clinical studies and targeted functional experiments.

CAMK2N1 is an endogenous cellular inhibitor of CAMK2 [42]. CAMK2 can regulate the cell cycle and is involved in tumorigenesis, invasion, and metastasis, and its expression can be identified in various cells and tissues, especially in the nervous system as well as in tumor tissues. It has been suggested that reducing CAMK2N1 in mice with essential hypertension lowers CAMK2 activity in the heart, leading to improved factors associated with CAD risk, such as hypertension, insulin resistance, left ventricular mass, and visceral obesity [43]. CAMK2N1 inhibits inflammatory activation and amplification in cardiomyocytes through the CaMKII*δ*‐p38/JNK‐NLRP3 inflammasome pathway, thereby significantly reducing ventricular remodeling and the occurrence of malignant arrhythmias in mice after myocardial infarction. Targeting the CAMK2N1 gene may be a potential therapeutic approach to improve CAD outcomes [44]. CAMK2N1 is implicated in CAD pathophysiology, potentially through its regulation of CaMKII activity and interconnected signaling pathways. Consequently, therapeutic targeting of CAMK2N1 holds promise for improving CAD prognosis.

In summary, C1QTNF3, PTGER2, and CAMK2N1 all play significant roles in the pathogenesis of CAD. Substantial evidence supports their potential as biomarkers for CAD diagnosis and prognosis. Our study uncovers a potential role for PRSS36 and B3GNT7 in the pathogenesis of CAD.

The PRSS36 gene is expressed in various tissues, including skeletal muscle, the liver, the heart, and other organs in the adult human body. The product of PRSS36 gene expression, Polyserase‐2, which comprises three structural serine protease domains, may play a role in hereditary diseases and tumorigenesis [45, 46]. In this study, the PRSS36 gene demonstrated modest diagnostic performance (AUC < 0.7), raising questions about its inclusion as a key gene. However, PRSS36 was consistently selected as a feature gene in both LASSO and SVM‐RFE analyses, indicating a robust and nonrandom association with CAD status. We contend that this consistent identification holds more fundamental significance in the discovery phase than any single diagnostic metric. Furthermore, this study is aimed at identifying novel BM‐related biomarkers for CAD, and PRSS36 remains poorly characterized in the cardiovascular field. Although its current diagnostic value is moderate, PRSS36 may reveal previously unrecognized aspects of CAD pathophysiology, offering substantial scientific value. As noted, the protease encoded by PRSS36 may participate in tissue remodeling during atherosclerosis, and its consistent downregulation in CAD patients suggests a potential protective role. While the role of PRSS36 as a potential biomarker in CAD remains underexplored, we hypothesize that its proteolytic function may represent an underlying mechanism influencing the disease, given its observed downregulation in CAD. The protein encoded by the B3GNT7 gene belongs to the *β*3GlcNAcT family, which is involved in glycosyl‐terminal LacNAc sulfation [47, 48]. B3GNT7 gene product promotes the synthesis of keratan sulfate proteins in the perineuronal region of the central nervous system [49] and is considered a biomarker of poor prognosis in various cancers [50]. As a glycosyltransferase, B3GNT7 may contribute to CAD by modulating cell surface glycosylation, thereby influencing key atherosclerotic processes such as leukocyte‐endothelial adhesion, inflammatory signaling, and lipoprotein retention. Its downregulation likely disrupts vascular homeostasis. The findings of this study suggest that these two genes may contribute to the pathogenesis of CAD through molecular mechanisms that remain to be fully elucidated. This discovery holds significant clinical implications and warrants further investigation.

In this study, we conducted an immune cell infiltration analysis and identified several correlations between key genes and different immune cell types. We observed significant differences in 11 different immune cell types between CTL and CAD. The most significant positive correlation was between CAMK2N1 and CD8 T cells, whereas the most significant negative correlation was between CAMK2N1 and monocytes. Current research demonstrates that CD8+ T cells in CAD patients show significant enrichment of the T‐cell receptor (TCR) signaling pathway [51], indicating their hyperactivated state. These cells predominantly localize in vulnerable atherosclerotic plaque regions—particularly the shoulder areas and fibrous caps where rupture frequently initiates. Notably, acute coronary syndrome lesions with intact fibrous caps exhibit marked CD8+ T‐cell infiltration accompanied by substantial release of cytotoxic mediators like Granzyme A and perforin, directly contributing to plaque destabilization [52]. Epidemiological evidence from a Chinese geriatric cohort further supports the clinical relevance of CD8+ T cells, showing an independent association between elevated CD4/CD8 ratios and CAD incidence [53]. In contrast, monocytes exhibit dual roles in CAD pathogenesis: While promoting disease through VCAM‐1‐mediated endothelial adhesion, transmigration into macrophages, ox‐LDL phagocytosis forming foam cells, and MMP secretion degrading extracellular matrix [54], they also demonstrate protective effects via secretion of proangiogenic factors (VEGF and FGF), modulation of myocardial fibrosis, and therapeutic potential evidenced by improved post‐MI cardiac function following bone marrow–derived monocyte infusion [55]. The differential correlations of CAMK2N1 with both CD8+ T cells and monocytes suggest its critical regulatory role in CAD‐related immunopathology. Our immune infiltration analysis provides computational insights into the alterations of the immune microenvironment in CAD. Although these immune cell proportions are inferred from transcriptomic data and require ultimate confirmation at the protein level by techniques such as flow cytometry or immunohistochemistry, the analysis proposes a specific and testable hypothesis that CAD progression may be closely linked to dynamic changes in immune populations, including CD8+ T cells and monocytes. Furthermore, the predicted correlation of the CAMK2N1 gene with these immune cells suggests its potential immunomodulatory role, thereby outlining a clear direction for future functional experiments.

Additionally, we predicted the TFs that regulate the key genes and constructed a regulatory network involving TF‐miRNA‐gene interactions. For instance, this study demonstrates that C1QTNF3 is coregulated by six TFs, such as PPARG. PPARG, a crucial member of the nuclear receptor superfamily of ligand‐dependent TFs, serves as a master regulator of adipocyte differentiation and metabolic homeostasis. In the pathological progression of CAD, PPARG demonstrates upregulated expression in arterial foam macrophages, implicating its regulatory role in atherogenesis [56]. Mechanistic investigations reveal that PPARG mediates antiatherogenic effects through precise modulation of reverse cholesterol transport, specifically by inhibiting foam cell formation and enhancing cholesterol efflux. However, the G allele of PPARG polymorphism rs10865710 may substantially attenuate PPARG transcriptional activity, consequently compromising its cholesterol efflux–promoting function and ultimately accelerating atherosclerotic plaque progression [57]. Although no direct interaction between C1QTNF3 and PPARG has been previously documented, our findings suggest that C1QTNF3 may be regulated by PPARG, thereby potentially influencing CAD pathogenesis and expanding the molecular mechanistic framework for the disease.

This study employed an integrative bioinformatics and experimental approach to identify five key BMRGs in CAD. These genes were consistently downregulated in CAD patients and demonstrated excellent diagnostic potential. Our findings provide clear directions for future clinical translation. First, these key genes exhibit preliminary potential as a candidate noninvasive blood‐based marker panel worthy of further validation for early screening and risk stratification of CAD. Compared to conventional computed tomography angiography (CTA), biomarkers offer key advantages for CAD management: They provide a noninvasive and convenient method for early disease warning and risk stratification, and they allow for repeatable measurements to dynamically monitor disease progression. This capability addresses the limitations of CTA in detecting molecular‐level pathology and facilitating frequent monitoring. Furthermore, the use of biomarkers opens new avenues for developing integrated diagnostic strategies that combine biomarkers with CTA. Second, the elucidated regulatory networks, such as the PPARG–C1QTNF3 axis, offer new candidates for targeted therapy development. Finally, this study outlines a blueprint for functional research, particularly for the previously uncharacterized roles of PRSS36 and B3GNT7 in CAD, whose specific mechanisms require further elucidation in cellular and animal models. In summary, our findings tightly link BM molecules to CAD pathogenesis, thereby deepening the mechanistic understanding of the disease and laying a mechanistic and theoretical foundation for novel diagnostic tools and targeted therapies.

Nevertheless, this study has certain limitations. The precise mechanistic roles of the key genes in CAD, as well as the immune infiltration results and regulatory networks, require further experimental validation. Owing to the limited sample size of the datasets used in this bioinformatics analysis, our findings may lack generalizability to the broader CAD population. Furthermore, the relatively small cohort may lead to limited statistical power, increasing the susceptibility to both false‐positive and false‐negative results. Therefore, validation in larger, independent cohorts is essential to confirm these preliminary findings. The generalizability of our conclusions is also constrained by the relatively small sample size used for qRT‐PCR validation. While this study confirmed the differential expression of key genes in CAD via qRT‐PCR, it did not extend to investigating their underlying regulatory mechanisms using in vitro cellular or in vivo animal models.

This study utilized two public datasets with significant tissue heterogeneity: GSE113079 (peripheral blood) and GSE125856 (adipose tissue). Tissue‐specific expression patterns may explain inconsistencies in the magnitude of differential expression and AUC values of some key genes between datasets. For instance, C1QTNF3 and B3GNT7 showed higher expression in blood than in adipose tissue, whereas PTGER2 and CAMK2N1 exhibited stable downregulation across both tissues, suggesting they may represent common molecular features of CAD. Nevertheless, the lack of direct lesion tissues (e.g., coronary plaques) means we cannot fully exclude bias from tissue‐specific expression. Future studies should include additional tissue types and larger cohorts to validate the generalizability of these findings.

To address these issues, we have outlined the following future work. We plan to systematically validate the functional roles of the key genes using in vitro siRNA knockdown/overexpression and in vivo animal models. Their interactions with immune cell subsets, such as CD8+ T cells and monocytes, will be examined via flow cytometry and immunofluorescence. Core regulatory relationships, including the PPARG–C1QTNF3 axis, will be verified using dual‐luciferase reporter and ChIP‐seq assays. Furthermore, we will expand our analysis to include larger datasets and extend our validation to a more homogeneous collection of clinical blood samples from well‐defined CAD subtypes—such as acute coronary syndrome or stable angina. This strategy will enhance the robustness of our findings and minimize confounding effects from sample heterogeneity.

**Nomenclature**
BMRGs: basement membrane–related genes
BMs: basement membranes
CAD: coronary artery disease
CTA: computed tomography angiography
CTL: control
DE‐BMRGs: differentially expressed basement membrane–related genes
DEGs: differentially expressed genes
EAT: epicardial adipose tissue
ECM: extracellular matrix
GEO: Gene Expression Omnibus
GO: Gene Ontology
GWAS: genome‐wide association study
KEGG: Kyoto Encyclopedia of Genes and Genomes
LASSO: least absolute shrinkage and selection operator
mAT: mediastinal adipose tissue
MMPs: matrix metalloproteinases
NADPH: nicotinamide adenine dinucleotide phosphate
PGE2: prostaglandin E2
PPP: pentose phosphate pathway
ROC: receiver operating characteristic
SAT: subcutaneous adipose tissue
SVM‐RFE: support vector machine–recursive feature elimination
TFs: transcription factors
WGCNA: weighted gene coexpression network analysis

## Author Contributions

Z.D., Y.Z., and Z.L. contributed to data collection and statistical analysis. Z.D. wrote the first draft of the manuscript. Y.Z. and Z.L. finished the figures. W.G. was responsible for project administration, funding acquisition, supervision, and writing—review and editing. All authors contributed to the critical revision of the final manuscript. Z.D., Y.Z., and Z.L. contributed equally to this work and should be regarded as co‐first authors.

## Funding

This work was supported by the National Natural Science Foundation of China (10.13039/501100001809, 82270259).

## Disclosure

All authors read and approved the final manuscript.

## Ethics Statement

Blood samples used for the purpose of verifying differential gene expression were approved by the Medical Ethics Committee of Xijing Hospital (Ethics Review Number: KY20232338‐C‐1).

## Consent

The authors have nothing to report.

## Conflicts of Interest

The authors declare no conflicts of interest.

## Supporting Information

Additional supporting information can be found online in the Supporting Information section.

## Supporting information


**Supporting Information 1** Table S1: Soft threshold selection statistics for WGCNA analysis. The table presents the scale‐free topology fitting index (SFT_R_sq) and mean connectivity values across soft threshold powers ranging from 1 to 20 in the GSE113079 dataset. A soft threshold power of *β* = 5 was selected as optimal, as it was the lowest value at which the scale‐free topology fit index (signed *R* ^2^) first exceeded 0.85 (*R* ^2^ = 0.852), while maintaining relatively high mean connectivity (61.06).

**Supporting Information 2** Figure S1: ROC curves of five key genes in the validation dataset GSE125856. Receiver operating characteristic (ROC) curves illustrating the diagnostic performance of C1QTNF3 (AUC = 0.648), PTGER2 (AUC = 0.708), CAMK2N1 (AUC = 0.775), PRSS36 (AUC = 0.750), and B3GNT7 (AUC = 0.699) in distinguishing CAD patients from controls in the GSE125856 adipose tissue dataset.

**Supporting Information 3** Figure S2: Construction and validation of the nomogram model in GSE125856. (A) Nomogram model constructed using five key genes for CAD risk prediction in the validation cohort. (B) Calibration curve assessing the predictive capability of the nomogram model (C − index = 0.8102). (C) Decision curve analysis (DCA) evaluating the clinical applicability of the nomogram model compared to individual genes. (D) Clinical impact curve demonstrating the model′s performance in predicting CAD risk across different high‐risk thresholds.

## Data Availability

The GSE113079 and GSE125856 datasets analyzed during the current study are available in the Gene Expression Omnibus (GEO) (https://www.ncbi.nlm.nih.gov/gds) repository (https://www.ncbi.nlm.nih.gov/geo/query/acc.cgi?acc=GSE113079; https://www.ncbi.nlm.nih.gov/geo/query/acc.cgi?acc=GSE125856). The original contributions presented in the study are included in the article/additional files; further inquiries can be directed to the corresponding author.
