# Supplementary figures and images for: Basement Membrane–Related Genes C1QTNF3, PTGER2, CAMK2N1, PRSS36, and B3GNT7: Novel Biomarkers for Coronary Artery Disease
Source: Cardiovasc Ther. 2026 Jul 9;2026:5358909. doi: 10.1155/cdr/5358909 (PMC13347310; doi:10.1155/cdr/5358909)

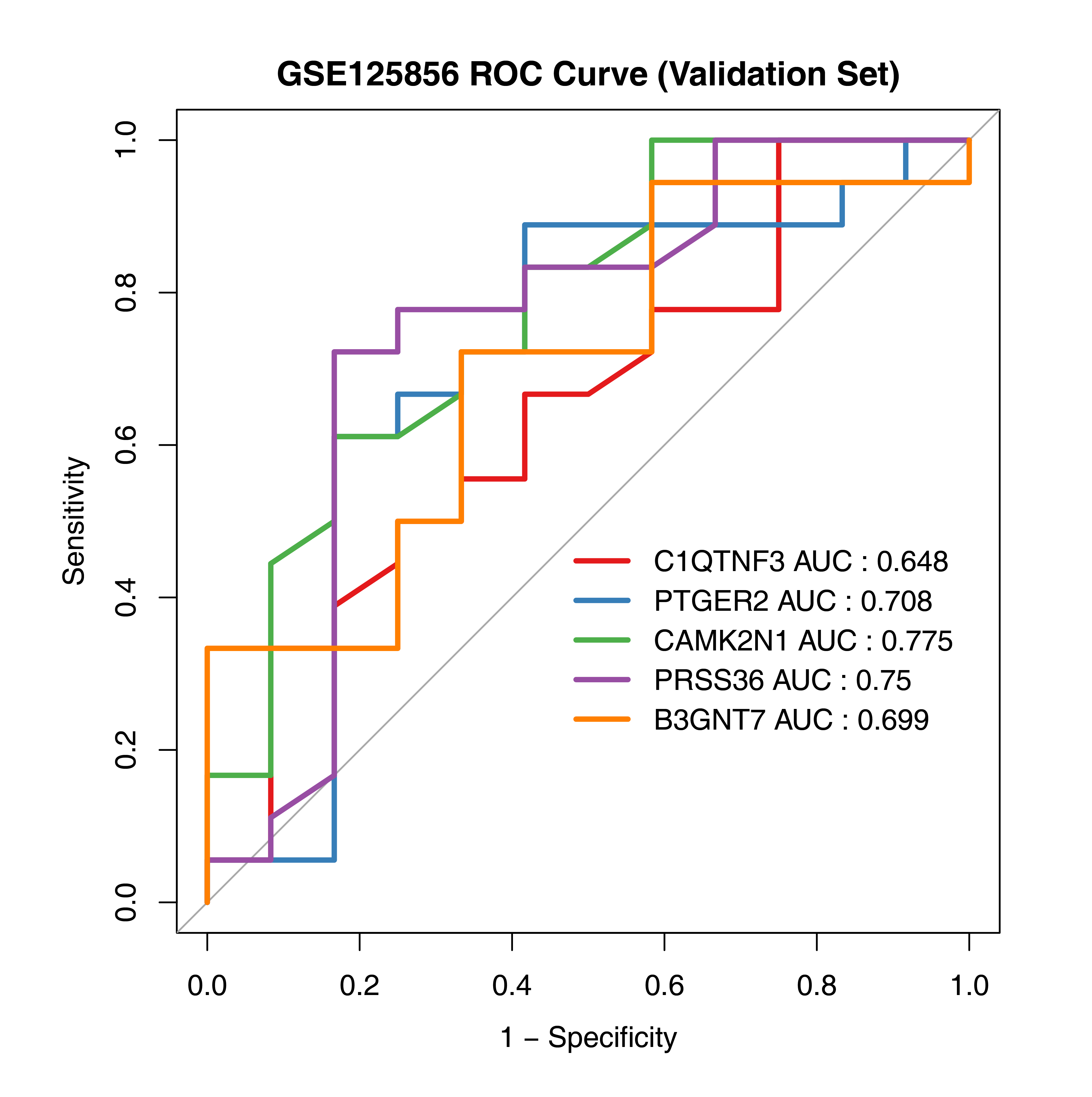

Supplement: Supplementary file 2 — Supporting Information 2 Figure S1: ROC curves of five key genes in the validation dataset GSE125856. Receiver operating characteristic (ROC) curves illustrating the diagnostic performance of C1QTNF3 (AUC = 0.648), PTGER2 (AUC = 0.708), CAMK2N1 (AUC = 0.775), PRSS36 (AUC = 0.750), and B3GNT7 (AUC = 0.699) in distinguishing CAD patients from controls in the GSE125856 adipose tissue dataset. [file CDR-2026-5358909-s001.tif]

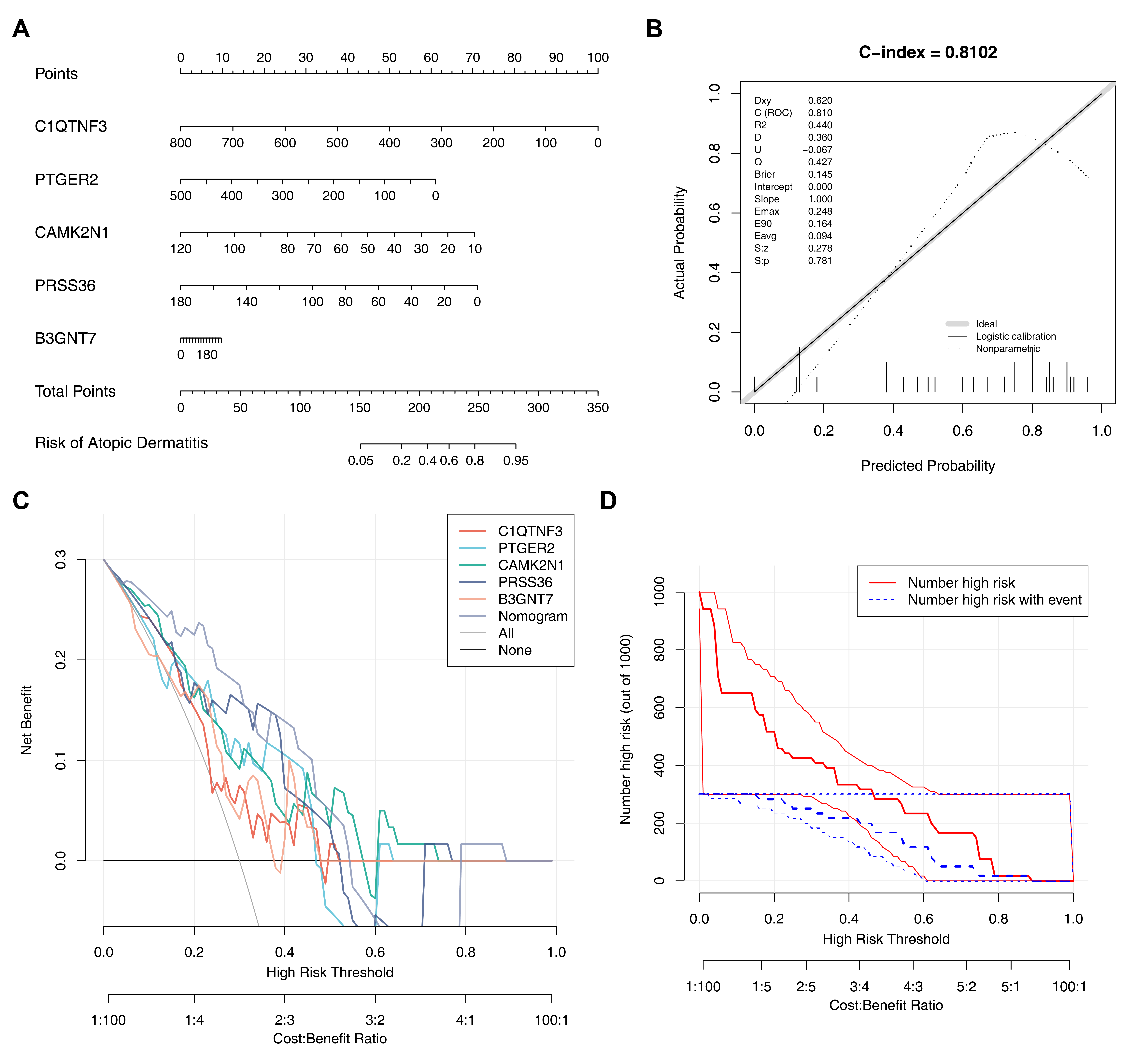

Supplement: Supplementary file 3 — Supporting Information 3 Figure S2: Construction and validation of the nomogram model in GSE125856. (A) Nomogram model constructed using five key genes for CAD risk prediction in the validation cohort. (B) Calibration curve assessing the predictive capability of the nomogram model (C − index = 0.8102). (C) Decision curve analysis (DCA) evaluating the clinical applicability of the nomogram model compared to individual genes. (D) Clinical impact curve demonstrating the model′s performance in predicting CAD risk across different high‐risk thresholds. [file CDR-2026-5358909-s002.tif]
